# When Goal Pursuit Gets Hairy: A Longitudinal Goal Study Examining the Role of Controlled Motivation and Action Crises in Predicting Changes in Hair Cortisol, Perceived Stress, Health, and Depression Symptoms

**DOI:** 10.1177/2167702621995214

**Published:** 2021-04-26

**Authors:** Anne Catherine Holding, Emily Moore, Amanda Moore, Jérémie Verner-Filion, Isabelle Ouellet-Morin, Richard Koestner

**Affiliations:** 1 Department of Psychology, McGill University; 2 Département des Sciences de l’Éducation, Université du Québec en Outaouais; 3 École de Criminologie, Université de Montréal

**Keywords:** action crisis, hair cortisol, motivation, goals, depression

## Abstract

The action crisis is a critical phase in goal striving during which the goal pursuer feels conflicted about persevering with the goal or initiating disengagement. Recent research suggests that goal motivation, specifically controlled motivation (i.e., pursuing a goal out of obligation and pressure), increases the likelihood of experiencing action crises. In turn, action crises in goal pursuit have been linked to increases in depression symptoms and cortisol. In the present 8-month longitudinal study, we tracked university students’ personal goals to examine whether the pursuit of controlled goals and the experience of action crises was associated with increasing levels of hair cortisol, perceived stress, poor health, and depression symptoms (*N* = 156). Structural equation modeling suggested that experiencing action crises in goal pursuit was associated with increases in markers of stress, depression, and ill-being. This effect was partially explained by controlled goal motivation. The clinical and theoretical implications of these findings are discussed.

The idiomatic phrase “getting hairy” refers to a situation becoming difficult, frightening, or complicated. Applied to goal pursuit, “getting hairy” could be one way to describe the experience of having an *action crisis*. An action crisis occurs when the pursuit of a goal is mired in setbacks and obstacles and one enters a prolonged state of decisional conflict, feeling torn between persevering with the goal and letting it go (Brandstätter et al., 2013). Preliminary research suggests that action crises are associated with increases in salivary cortisol (Brandstätter et al., 2013) and worsening symptoms of depression (Holding et al., 2017), highlighting the clinical relevance of investigating this phase in goal striving. In addition, recent research suggests that the pursuit of controlled goals (i.e., the pursuit of goals out of feelings of internal or external pressure) makes individuals more vulnerable to the experience of action crisis during goal pursuit (Holding et al., 2017). However, little is known about the long-term impact of controlled goal pursuit and action crises for stress and physical and mental health. To this end, we studied goal pursuit “getting hairy” in the second, literal sense of the phrase: We sampled participants’ hair to measure the chronic secretion of the stress hormone cortisol as they pursued three personal goals over the span of 8 months. Cortisol is a hormonal product of the reactivity of the limbic hypothalamic-pituitary-adrenal (HPA) axis that signals the activation of a stress-induced coping process (Dickerson & Kemeny, 2004). Whereas the physiological changes brought about by a stress response help the body to maintain homeostasis in the face of a stressor, sustained responses or repeated reactivations are hypothesized to wear and tear the stress system, with detrimental consequence for mental and physical health (Staufenbiel et al., 2013). Unlike measuring cortisol using saliva or blood, the collection of hair for cortisol assessment allows researchers to capture systemic differences in cortisol secretion over the preceding 3 months and is less affected by time-varying confounders than other biospecimens (Russell et al., 2012).

To the best of our knowledge, hair cortisol has not been used in longitudinal health and goal research. Our aim was thus to examine how controlled motives and the experience of action crises during goal pursuit affected changes in hair cortisol as well as perceived stress, health, and depression symptoms.

## Clinical Outcomes Associated With Action Crises in Goal Pursuit

The pursuit of personal goals lends structure and meaning to peoples’ lives and is a vital source of sustained well-being (Ryan & Deci, 2017). However, when goal pursuit becomes overly costly and demanding, persevering with a problematic goal can have negative consequences for mental and physical health (Wrosch et al., 2013). In this context, goal disengagement is adaptive, allowing the pursuer to conserve motivational resources and avoid repeated experiences of failure (Wrosch et al., 2013). However, individuals are often reluctant to part with goals even when chances of success are low (Sleesman et al., 2012). Instead, individuals are likely to enter a phase of action crisis, during which they feel deeply conflicted about investing further or cutting losses and initiating disengagement (Brandstätter et al., 2013).

Given the uncertainty and conflict associated with action crises, this phase in goal striving is experienced as highly unpleasant and is associated with decreased subjective well-being (Brandstätter et al., 2013) as well as increased symptoms of depression (Holding et al., 2017). Moreover, action crises in goal striving have been associated with physiological consequences, including compromised recovery during physical therapy (Wolf et al., 2019), somatic symptoms, poorer physical performance, as well as markers of increased physiological stress, such as steeper salivary cortisol slopes in runners (Brandstätter et al., 2013). Together, these findings suggest that being in a prolonged or severe action crisis may compromise well-being and physical health.

## Controlled Motivation in Goal Pursuit

In addition, we considered whether goal motivation, which is an antecedent of the action crisis, also relates to changes in markers of stress and ill-being over time. According to self-determination theory (SDT; Ryan & Deci, 2017), a leading theory of human motivation, controlled motivation for goals is characterized by a sense of internal or external pressure to act, which often alienates one from one’s own priorities and values. Thus, even when controlled goals are self-generated, they may not feel truly “personal” given the external incentives (e.g., obtaining rewards or pleasing others—*external regulation*) or internal pressures (e.g., reducing feelings of guilt and shame—*introjection*; Sheldon & Elliot, 1998). For example, someone could set a goal to start exercising because their partner told them to (external) or because they feel ashamed about their weight (introjected). Both external regulation and introjection are considered controlled forms of motivation (Ryan & Deci, 2017). Controlled motivation is contrasted with autonomous motivation, which involves pursuing a goal because it is interesting and fun (*intrinsic motivation)* or personally important and meaningful (*identified motivation*).

Controlled motivation has recently been shown to predict the occurrence of more severe action crises during goal pursuit (Holding et al., 2017). A likely explanation is that controlled goals are less representative of enduring interests and values and are only partially or superficially integrated into the self (Ryan & Deci, 2017). This makes controlled goals more susceptible to inner conflict and feelings of ambivalence when obstacles or setbacks in goal pursuit arise, not least because controlled goals feel more strenuous to pursue (Werner et al., 2016) and are more susceptible to competing desires and temptations (Milyavskaya et al., 2015). Together, these factors make controlled goals more prone to action crises. A complementary theoretical account was offered by Herrmann and Brandstätter (2013), who found that goals lower in self-concordance (e.g., low autonomous and high controlled motivation) were at greater risk of action crises, likely because of reduced goal shielding.

Emerging evidence suggests that pursuing goals for controlled reasons may come at a cost to the pursuers’ mental health. For example, Holding et al. (2017) observed that the pursuit of controlled personal goals led to increased symptoms of depression over the course of an academic semester, an effect mediated by action-crisis severity. Likewise, a longitudinal study examining career goal pursuit in young adults found that pursuing a career goal for controlled motives was associated with increased psychological distress (Holding et al., 2020).

We sought to extend this work by studying the emotional and physical toll of controlled goal pursuit on longitudinal changes in markers of physiological chronic stress and ill-being. To date, only one experimental study has linked SDT’s concept of “feeling controlled” to increases in cortisol. Specifically, Reeve and Tseng (2011) experimentally examined whether being exposed to a controlling teacher was associated with increased salivary cortisol and found that students’ salivary cortisol increased as a function of the teacher’s controlling motivational style during a 20-min puzzle-solving task.

## The Present Work

To evaluate whether controlled goal motivation and experiences of action crises during goal pursuit affected changes in stress and ill-being, we chose a prospective longitudinal design and assessed a marker of chronic cortisol elevation among other self-reported markers of stress and ill-being as participants pursued three personal goals over the course of an academic year. This is different to previous research that has considered only the short-term consequences of controlled motivation and action crises on salivary cortisol (e.g., Brandstätter et al., 2013; Reeve & Tseng, 2011). Recent research suggests that saliva and blood cortisol biospecimens are best suited to measure responses to acute stressors but are not ideally suited for tracking change in persistent differences of cortisol secretion over time (Russell et al., 2012). Because the effects of action crises in goal pursuit are thought to unfold over the course of several weeks and months, the collection of hair samples for cortisol measurement may more closely signal the changes in cortisol levels to which the brain is exposed over an extended period of time compared with biospecimens that capture acute cortisol secretion (Kirschbaum et al., 2009). Indicators of stress and ill-being were assessed at the beginning and end of the academic year. Action-crisis severity was measured midway through the academic year, the point at which previous research has documented that action crises are likely to set in (Brandstätter et al., 2013). We then proceeded to test our hypothesis that controlled motives for goals would bring about more severe action crisis in goal pursuit and would indirectly predict increases in markers of stress and ill-being.

## Method

### Participants

The study sample consisted of 156 university students (89% female; 57% White, 28% Asian, 4% Hispanic), 17 to 38 years old (*M* = 19.68, *SD* = 2.41), recruited at a large public North American university. To be eligible for the hair sampling, participants needed a hair length of minimum 3 cm and could not have hair that was dyed, permed, or bleached.

### Procedure

Participants were recruited through posters to take part in a longitudinal study on personal goals and well-being. Participants had the option of just enrolling in the longitudinal survey study or also having their hair sampled for cortisol at both the beginning and at the end of the academic year. The sample included in this report represents only those participants who agreed to have their hair sampled for cortisol measurement.

At the beginning of the academic year in September (Time 1 [T1]), participants were sent an online survey in which they identified three personal goals they planned to pursue over the course of the academic year. At this time, they also rated their motivation for each goal as well as their perceived stress, health symptoms, and symptoms of depression. Approximately 4 to 6 weeks later, they visited our lab for hair collection conducted by trained graduate students who followed the sampling guidelines outlined by Ouellet-Morin and colleagues (2016). This timeline was intended so that the middle point of the measure of chronic cortisol—which captures the previous 3 months of stress—would align with the T1 survey. Three months later, at the end of the academic semester in mid-December (Time 2 [T2]), participants completed a follow-up survey in which action-crisis severity for each goal was assessed. At the end of the academic year in April (Time 3 [T3]), participants responded once more to the questions about their perceived stress, health symptoms, and symptoms of depression, and they returned to the lab for a second hair sample. This study was approved by the research ethics board of the university, and participants were compensated $75 for their participation in this study. The attrition rate was low (T2 survey = 7%; T3 survey = 14%). Of the 156 participants who completed the first hair sampling, 90% returned for the second hair sampling in the following academic semester.

### Measures

#### Goal selection

Participants were prompted to nominate three goals they planned to pursue over the academic year using the instructions adapted from Sheldon and Kasser (1998). Examples of the goals that students generated were “I want to raise my GPA to a 3.5” and “I want to get back to a normal weight.”

#### Controlled goal motivation

At T1, participants rated the extent to which they were pursuing each goal for external reasons (because somebody else wants you to or because you’ll get something from somebody if you do) and introjected reasons (because you would feel ashamed, guilty, or anxious if you didn’t—you feel that you ought to strive for this) on a 7-point Likert scale ranging from 1 (*strongly disagree*) to 7 (*strongly agree*) using the items assessed by Sheldon and Kasser (1998). In line with previous research, external and introjected reasons were averaged to compute controlled motivation (Koestner et al., 2008). Controlled motivation was averaged across the three personal goals (α = .68).

#### Action crisis

At T2, we administered the six-item Action Crisis Scale for each goal to assess action-crisis severity (Brandstätter et al., 2013) using a validated English version of the scale (Holding et al., 2017). A sample item is “Lately I feel torn between continuing to strive for this goal and abandoning it.” Participants rated the items on a 7-point scale from 1 (*strongly disagree*) to 7 (*strongly agree*). The indicators of internal consistency were acceptable for each goal (average α = .78). Action-crisis severity was averaged across the three personal goals.

#### Symptoms of depression

We administered the Center for Epidemiologic Studies Depression Scale (Radloff, 1977) to assess symptoms of depression at T1 and T3. The scale includes 10 items such as “I could not get going” and is measured on a 4-point Likert scale ranging from 0 (*rarely or none of the time*) to 3 (*most or all the time*) with the preceding 2 weeks as a frame of reference. The depression symptoms score was computed by adding the items. Alphas were acceptable for both T1 (α = .73) and T3 (α = .84).

#### Perceived Stress Scale

The participants reported their subjective stress by completing the Perceived Stress Scale (PSS; Cohen et al., 1983) at T1 and T3. The PSS consists of 10 items such as “How often have you felt difficulties were piling up so high that you could not overcome them?” on a scale from 0 (*never*) to 4 (*very often*) with the preceding 2 weeks as a frame of reference. Four items were reverse-scored. αs were acceptable for both T1 (α = .85) and T3 (α = .87).

#### Hair cortisol

Hair samples were collected at T1 and T3 for cortisol measurement. Trained graduate students cut hair strands of approximately 1 cm width from the posterior vertex of participants’ heads near the scalp. The first 3-cm section of each hair sample was analyzed in a single batch at a specialized lab following the protocol outlined in Ouellet-Morin et al. (2016). To derive an indicator of cortisol change over the academic year, we estimated the standardized residual of each participant’s cortisol levels at T3, according to the levels noted at the beginning of the academic year (i.e., T1). Accordingly, participants served as their own baseline control with regard to the cortisol measurement, which allowed us to indirectly control for a host of unmeasured confounders. Hair-cortisol concentrations ranged from 3.63 to 137.94 pg/mg in the first sample (T1: *M* = 18.91, *SD* = 14.35) and from 4.66 to 106.33 pg/mg in the second sample (T2: *M* = 15.36, *SD* = 12.04). These values were similar in range to values obtained in other hair-cortisol studies (Ouellet-Morin et al., 2016).

#### Physical Health Questionnaire

To measure subjects’ perceived physical health, we asked participants to complete the 12-item Physical Health Questionnaire at T1 and T3 (Schat et al., 2005). Participants responded to questions such as “How often have you suffered from upset stomach (indigestion)?” on a Likert scale ranging from 1 (*not at all*) to 7 (*almost all of the time*), with the preceding 2 weeks as a frame of reference. αs were acceptable for both T1 (α = .85) and T3 (α = .85).

#### Hair-related and sociodemographic factors

Information about the natural state of the hair (e.g., color, curvature) and usual care (e.g., frequency of washing, hair treatments) was obtained through a short questionnaire given to the participants. Participants were also asked about sociodemographic and lifestyle factors, including sex, age, and body mass index (BMI).

## Results

### Preliminary results

The descriptive information of our key variables as well as the correlation estimates between these variables are presented in Table 1. Students’ perceived stress did not significantly change over the course of the academic year (T1: *M* = 1.97, *SD* = 0.66; T3: *M* = 2.01, *SD* = 0.67), *t*(134) = 0.06, *p* = .40. However, students’ health symptoms increased over the course of the academic year, from T1 (*M* = 2.85, *SD* = 0.99) to T3 (*M* = 3.01, *SD* = 1.06), *t*(134) = −2.94, *p* = .004. Likewise, students reported significantly greater symptoms of depression at T3 (*M* = 12.02, *SD* = 5.73) compared with T1 (*M* = 10.66, *SD* = 4.66), *t*(133) = −3.53, *p* = .001. Students showed decreasing levels of chronic cortisol secretion over the year (T1: *M* = 18.40, *SD* = 14.42; T3: *M* = 15.36, *SD* = 12.04), *t*(140) = 3.41, *p* = .001, despite the retest stability of this measure over time, *r*(141) = .69, *p* < .001.

**Table 1.** Descriptive Information and Correlations Between Study Variables

Variable	1	2	3	4	5	*M*	*SD*
1. Mean controlled motivation for personal goals T1	—					3.12	1.14
2. Mean action-crisis severity on personal goals T2	.20*	—				3.76	0.81
3. Change in hair cortisol T1 to T3	−.06	.20*	—			0.00	1.00
4. Change in perceived stress T1 to T3	.02	.30***	.12	—		0.02	0.98
5. Change in health symptoms T1 to T3	.04	.23**	.19*	.38***	—	0.00	1.01
6. Change in depression symptoms T1 to T3	.00	.36***	.13	.62***	.45	−0.07	0.93

Note: T1 = Time 1; T2 = Time 2; T3 = Time 3.

* *p* < .05. **p < .01. ***p < .001.

Preliminary analyses showed that baseline cortisol concentrations did not differ between men and women. Moreover, change in chronic cortisol secretion was not significantly associated with the participants’ gender, age, hair type, frequency of hair washing and conditioning, hair treatments, BMI at T1, BMI at T3, or the change in BMI from T1 to T3. As can be seen in the bivariate associations presented in Table 1, controlled motivation for personal goals was positively related to midyear action-crisis severity. Midyear action-crisis severity was also positively associated with temporal changes noted on all four indicators of stress and health over the academic year. Change toward increased hair cortisol was positively correlated with the composite score of increases in all three subjective indicators of ill-being (*r* = .18, *p* = .04).

### Structural equation model

**Fig. 1.**
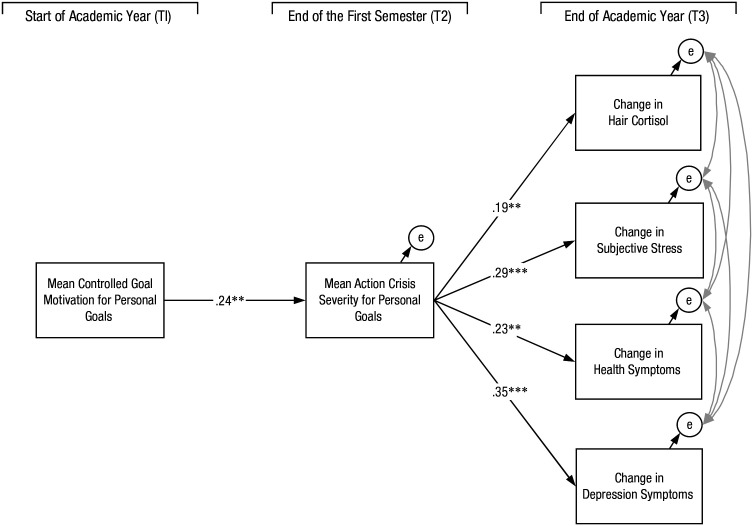
The final model of the results of the structural equation model. Covariances were specified for the outcome that are not drawn in the figure. Asterisks indicate significant variables (**p < .01, ***p < .001).

Structural equation modeling (SEM) was performed to test the role of controlled motivation for goal pursuit on action-crisis severity and the role of action-crisis severity for predicting changes in hair cortisol, perceived stress, health symptoms, and symptoms of depression across the academic year (see Fig. 1). We computed standardized residual change scores for all the markers of stress and ill-being from baseline to T3. The robust maximum likelihood estimation (MLR) procedures offered within MPlus (Version 7.3; Muthén & Muthén, 2012) were used to perform the SEM because this method is robust to potential deviations in normality. The following fit indices were thus given priority in model evaluation: comparative fit index (CFI), root mean square error of approximation (RMSEA), and standardized root mean square residual (SRMR). For acceptable model fit, the CFI should be .90 or higher, and the RMSEA and SRMR should be 0.06 or lower (Kline, 2011).

Results revealed that controlled motives for goals set at the beginning of the academic year were positively related to midyear action-crisis severity (β = 0.24 *SE* = 0.09, 95% confidence interval [CI] = [0.07, 0.40]). Moreover, midyear action-crisis severity was positively associated with change in hair cortisol^1^ (β = 0.19, *SE* = 0.07, 95% CI = [0.04, 0.32]), change in perceived stress (β = 0.29, *SE* = 0.09, 95% CI = [0.11, 0.46]), change in health symptoms (β = 0.23, *SE* = 0.09, 95% CI = [.05, .41]), and change in depression symptoms (β = 0.35, *SE* = 0.08, 95% CI = [0.18, 0.50) from baseline to end of the academic year. Note that all the indirect paths were significant: hair cortisol: β = 0.05, *SE* = 0.02, 95% CI = [0.01, 0.11]; perceived stress: β = 0.07, *SE* = 0.07, 95% CI = [0.02, .013]; health symptoms: β = 0.06, *SE* = 0.03, 95% CI = [0.01, 0.13]; depression symptoms: β = 0.09, *SE* = 0.04, 95% CI = [0.02, 0.14]. These results support the mediating role of action-crisis severity in explaining the indirect associations between controlled motivation for personal goals and changes in stress and health outcomes over the academic year. Overall, the proposed model had an acceptable fit to the data, MLR: χ^2^(*df* = 4) = 3.25, *p* = .52, CFI = 1.00, RMSEA = 0.00 (0.00, 0.11), SRMR = 0.03.

## Discussion

The goal of the present study was to examine whether controlled motivation for personal goals and conflicted goal striving would be associated with changes in stress levels, depression symptoms, and poor health symptoms over time. In line with our hypothesis, we found that experiences of action crises in personal goal pursuit were associated with increases in a measure of physiological stress (i.e., hair cortisol), perceived stress, and health and depression symptoms across the academic year. This is the first study to examine the long-term impact of action crises across a wide range of indicators of stress and health. It is noteworthy that an asset of this study was the repeated assessment of an objective biological indicator of stress. Replicating the results of Holding et al. (2017), we found that controlled motives for goal pursuit were positively associated with action-crisis severity and increases in depressive symptoms. We also extended these findings by showing that controlled motivation for personal goals was indirectly associated with increases in markers of stress and ill-being via the development of action crises.

The current findings highlight the clinically relevant implications of experiencing action crises in personal goal pursuit. Personal goals play a huge role in people’s everyday lives, and understanding the associations between the experience of goal-related conflict and indicators of stress and health over time underscores the importance of considering action crises as part of a more integrated model of health. To date, the majority of research linking goal failure to depression and worsening health has focused on how difficulties with *disengagement* from unattainable goals can have adverse effects on individuals’ biological functioning and adaptation (Wrosch et al., 2013). Our findings suggest that the phase in goal striving that precedes goal disengagement (i.e., the action crisis) may be equally costly to individuals in terms of their health. Indeed, future research is needed to explore whether difficulties disengaging from unattainable goals are maladaptive precisely because the individual experiences repeated and ongoing action crises. This offers an intriguing avenue for future research to investigate how clinicians can help clients reappraise problematic personal goals and effectively resolve action crises.

Our study also highlights the potential risks of controlled goal pursuit, which appears to indirectly increase markers of stress and ill-being through the development of action crises. This finding adds to an emerging body of research documenting the potential harms of feeling controlled about pursuing personal goals (Holding et al., 2017, 2020). Because effective goal setting and striving are central to many forms of psychotherapy, this study underlines the importance for clinicians to stay attuned to clients’ expression of controlled motivation for goal pursuit as well as support clients’ internalization of autonomous motives for personal goals (see Ryan & Deci, 2017). Moreover, these finding have implications for SDT, which has a history of being focused on positive and humanistic aspects of human functioning. Indeed, there have been two recent movements within self-determination theory (SDT) (a) to study the mechanisms that bring about both optimal, positive functioning and nonoptimal, pathological functioning and (b) to consider the biological underpinnings of the motivational process it describes (Ryan & Deci, 2017). The present study advances SDT research in line with both of these endeavors by indirectly linking controlled motivation for personal goals with markers of pathological functioning (i.e., increased symptoms of depression) and a biological marker of chronic stress through the experience of action crises.

Finally, our use of hair-cortisol sampling for longitudinal goal research represents an important methodological contribution and strength of the article. This study highlights the feasibility of conducting repeated hair-cortisol sampling within the context of a longitudinal study on goals, health, and ill-being. The attrition rate was low, and the sampling procedure was well tolerated. Moreover, because the chronicity of the burden associated with action crises was central to the study, hair cortisol was an ideal indicator of long-term HPA axis functioning. Indeed, to date, most research on goal-related difficulties and cortisol secretion has been cross-sectional and has used short-term markers of cortisol secretion. Given that chronic cortisol elevation appears to be both a pathway to worsening mental health as well as a biological marker that can be used to distinguish clinical from nonclinical populations (Staufenbiel et al., 2013), hair-cortisol sampling may be an invaluable tool in clinical psychology research for tracking chronic cortisol levels in at-risk populations or in intervention work.

This study was not without limitations. Although the longitudinal design, repeated assessments of clinically relevant outcomes, and assessment of an objective indicator of stress represent strengths of this study, additional measurement times would have facilitated a more fine-tuned analysis of the temporal interplay between these variables. Note that the correlational design does not allow us to draw causal conclusions. Our predominantly female sample may have restricted our power to observe gender effects. In addition, replicating the results in a clinical sample would enhance the generalizability and clinical utility of the present results and support examining whether underlying clinical disorders, such as major depression, increase people’s vulnerability to setting controlled goals or experiencing action crises in goal pursuit.

In conclusion, the present research contributes to clinical psychological science by identifying controlled goal motivation and action crises in goal pursuit as factors that contribute to increases in chronic stress, symptoms of depression, and poor health.

## Transparency

*Action Editor:* Erin B. Tone

*Editor:* Kenneth J. Sher

*Author Contributions*

A. C. Holding developed the study concept. I. Ouellet-Morin and R. Koestner contributed to the study design. Testing and data collection were performed by A. C. Holding, E. Moore, A. Moore, and J. Verner-Filion. A. C. Holding and J. Verner-Filion performed the data analysis and interpretation under the supervision of R. Koestner and with recommendations from I. Ouellet-Morin. A. C. Holding drafted the manuscript, and all of the authors provided reviews. All of the authors approved the final manuscript for submission.

## Notes

1. The same pattern of results is obtained when we winsorize the hair cortisol standardized residual.
